# Aptamer-modified magnetic nanoprobe for molecular MR imaging of VEGFR2 on angiogenic vasculature

**DOI:** 10.1186/1556-276X-8-399

**Published:** 2013-09-26

**Authors:** Bongjune Kim, Jaemoon Yang, Myeonghwan Hwang, Jihye Choi, Hyun-Ouk Kim, Eunji Jang, Jung Hwan Lee, Sung-Ho Ryu, Jin-Suck Suh, Yong-Min Huh, Seungjoo Haam

**Affiliations:** 1 Department of Chemical and Biomolecular Engineering, College of Engineering, Yonsei University, Seoul 120-749, Republic of Korea; 2 Department of Radiology, College of Medicine, Yonsei University, Seoul 120-752, Republic of Korea; 3 POSTECH Aptamer Initiative Program, Division of Integrative Bioscience and Biotechnology, Pohang University of Science and Technology, Pohang 790-784, Republic of Korea

**Keywords:** Magnetic nanocrystal, Aptamer, VEGFR2, Angiogenesis, Magnetic resonance imaging, Molecular imaging

## Abstract

Nucleic acid-based aptamers have been developed for the specific delivery of diagnostic nanoprobes. Here, we introduce a new class of smart imaging nanoprobe, which is based on hybridization of a magnetic nanocrystal with a specific aptamer for specific detection of the angiogenic vasculature of glioblastoma via magnetic resonance (MR) imaging. The magnetic nanocrystal imaging core was synthesized using the thermal decomposition method and enveloped by carboxyl polysorbate 80 for water solubilization and conjugation of the targeting moiety. Subsequently, the surface of the carboxylated magnetic nanocrystal was modified with amine-functionalized aptamers that specifically bind to the vascular growth factor receptor 2 (VEGFR2) that is overexpressed on angiogenic vessels. To assess the targeted imaging potential of the aptamer-conjugated magnetic nanocrystal for VEGFR2 markers, the magnetic properties and MR imaging sensitivity were investigated using the orthotopic glioblastoma mouse model. In *in vivo* tests, the aptamer-conjugated magnetic nanocrystal effectively targeted VEGFR2 and demonstrated excellent MR imaging sensitivity with no cytotoxicity.

## Background

Magnetic resonance (MR) imaging is a superior molecular imaging technique for clinical diagnosis of cancer because it provides noninvasive tomographic imaging with high spatial resolution [1,2]. The sensitivity of MR imaging has significantly improved in recent years by using magnetic nanocrystal (MNC) because an enhanced *T*2 shortening effect is ascribed to the high crystallinity of MNC [3-5]. In particular, the immobilization of a targeting moiety on the magnetic nanocrystal has facilitated biomarker-specific molecular imaging by MR [6]. Thus, biomarker-specific molecular imaging for cancer enables early and specific detection of cancer cells and facilitates analysis of disease progression to improve the survival rate of cancer patients [7,8].

Glioblastoma is the most common and lethal intracranial tumor. This brain cancer exhibits a relentless malignant progression with characteristics of widespread invasion, destruction of normal brain tissue, resistance to conventional therapeutic approaches, and certain death. In addition, glioblastoma is among the most highly vascular of all solid tumors. Although there are marked genomic differences between primary (*de novo* pathway) and secondary (progressive pathway) glioblastoma, a physiological adaptation to hypoxia and critical genetic mutations commonly converge on a final tumor angiogenesis pathway. Therefore, precise molecular imaging of glioblastoma can be a crucial step for effective treatment [9,10]. Recent studies have identified key angiogenic factors, such as basic fibroblast growth factor, interleukin-8, hypoxia-inducible factors, and vascular endothelial growth factor A (VEGFA). Among these, VEGFA and one of its receptors (vascular endothelial growth factor receptor 2, VEGFR2) have been established as the primary proangiogenic factors [11,12].

**Figure 1:**
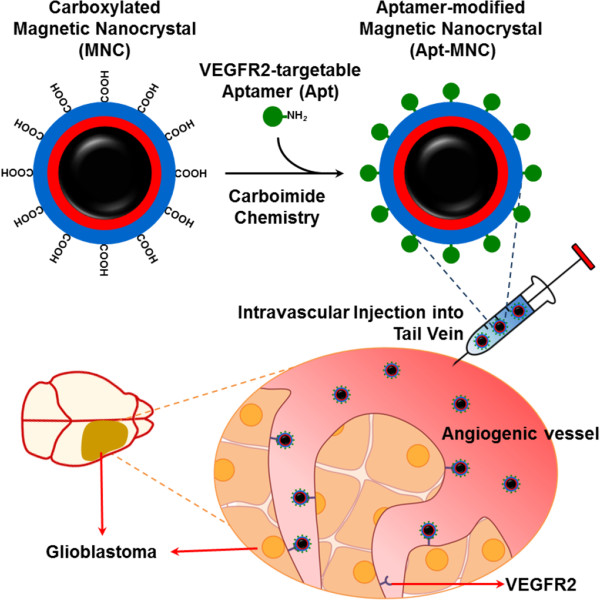
**Schematic illustration of the preparation steps for VEGFR2-specific magnetic nanoprobe.** Schematic illustration of the preparation steps for VEGFR2-specific magnetic nanoprobe and application for MR imaging of angiogenic vasculature from glioblastoma.

In this study, we developed a VEGFR2-targetable MR imaging probe to enable precise recognition of angiogenic vasculature of glioblastoma. To synthesize a sensitive MR imaging contrast agent, monodispersed MNC (Fe_3_O_4_) with high crystallinity was synthesized by thermal decomposition method and subsequently enveloped with tri-armed carboxyl polysorbate 80 by a nanoemulsion method. To prepare the magnetic nanoprobe for specific binding with VEGFR2 on angiogenic vessels, VEGFR2-specific aptamers (Apt) based on nucleic acid were immobilized on the surface of carboxylated MNC. Recently, Apt based on single-stranded nucleic acid molecules have been developed as a targeting moiety due to their high affinity and selectivity for a variety of chemical and biological molecules [13]. The main advantage of Apt over antibody lies in the relative ease with which they can be selected for analysis of any target and their stability against biodegradation and denaturation [14]. The chemical structure and colloidal size of aptamer-modified MNC (Apt-MNC) were evaluated. To assess the molecular imaging potential of Apt-MNC, we investigated MR imaging sensitivity and binding affinity for angiogenic vessels expressing VEGFR2 using the orthotopic glioblastoma mouse model. A conceptual schematic illustration is provided in Figure 1.

## Methods

### Materials

Iron (III) acetylacetonate, 1,2-hexadecanediol, oleic acid, oleylamine, benzyl ether, polysorbate 80, succinic anhydride, 4-dimethylaminopyridine, triethylamine, and 1,4-dioxane were purchased from Sigma-Aldrich. The anti-VEGFR2 DNA aptamer [51-mer sequence: H_2_N-C6-5′-d(ACGAGCZACG ACGZCZGGZG ZAAZZZAZAA AGACACZGZG ZAZAZCA ACAA)-3′; Z is 5-*N*-(benzylcarboxyamide)-2′-deoxyuridine (BzdU), with MW 17,567.05 Da] can target VEGFR2. This anti-VEGFR2 DNA aptamer (Cat number 186, Kd = 0.12 nM) was kindly provided by Aptamer Science, Inc. (http://www.aptsci.com/product/product.tml). Phosphate-buffered saline (PBS; 10 mM, pH 7.4), Dulbecco's modified Eagle medium (DMEM), and minimal essential medium (MEM) were purchased from Gibco (Life Technologies Corporation, Carlsbad, CA, USA). All other chemicals and reagents were analytical grade and obtained from Sigma-Aldrich (St. Louis, MO, USA).

### Synthesis of carboxylated magnetic nanocrystal

As described previously, we synthesized monodispersed MNC by the thermal decomposition method. In detail, 2 mmol of iron (III) acetylacetonate, 10 mmol of 1,2-hexadecanediol, 6 mmol of oleic acid, and 6 mmol of oleylamine were dissolved in 20 mL of benzyl ether in an ambient nitrogen atmosphere. The mixture was pre-heated to 200°C for 2 h and refluxed at 300°C for 30 min. The resulting solution containing MNC was cooled to room temperature, and MNC was purified with an excess of pure ethanol. The synthesized MNC was grown to a size of 12 nm by a seed-mediated growth method [15]. To immobilize VEGFR2-specifc aptamers on MNC, carboxylated MNC was fabricated using tri-armed carboxyl polysorbate 80 by a nanoemulsion method. Here, the terminal group of polysorbate 80 was modified with carboxyl group using succinic anhydride to provide the conjugation site for aminated aptamers [16], by adding 4 mL of n-hexane containing 10 mg of MNC to 20 mL deionized water containing 100 mg carboxyl polysorbate 80. After mutual saturation of the organic and aqueous phases, the mixture was sonicated for 20 min at 190 W with vigorous stirring. After the sonication, the organic solvent was evaporated rapidly using a rotary evaporator to form carboxylated MNC, and free molecules were removed using a centrifugal filter (Centriprep YM-3, 3,000 Da cutoff, Amicon, Millipore, Billerica, MA, USA) [17].

### Preparation of VEGFR2-targetable aptamer-conjugated magnetic nanoprobe

VEGFR2-specific aptamers were conjugated with carboxylated MNC for specific imaging of VEGFR2 in glioblastoma tumors via MR imaging. In detail, 38 μmol of 1-ethyl-3-(3-dimethylaminopropyl)-carbodiimide, 38 μmol of sulfo-*N*-hydroxysuccinimide, and 11 nmol of aptamers were added to 10 mg of carboxylated MNC suspended in 5 mL of nuclease-free water. After the reaction at 4°C for 24 h, Apt-MNC was purified with an ultracentrifugal filter (Amicon Ultra; Millipore, Billerica, MA, USA) to remove side-products [18].

### Characterization of Apt-MNC

The characteristic bands for polysorbate 80 and carboxyl polysorbate 80 were analyzed using Fourier transform infrared (FTIR) spectroscopy (Excalibur Series, Varian, Inc., Palo Alto, CA, USA). The size and morphology of Apt-MNC were investigated using transmission electron microscopy (TEM, JEM-2100 LAB6, JEOL Ltd., Akishima, Tokyo, Japan). The hydrodynamic diameter and surface charge of carboxylated MNC and Apt-MNC were measured using laser scattering (ELSZ, Otsuka Electronics, Hirakata, Osaka, Japan). The magnetic hysteresis loop and the saturation magnetization of Apt-MNC were measured in dried sample at room temperature using a vibrating sample magnetometer (model-7300, Lake Shore Cryotonics Inc., Westerville, OH, USA). The *T*2-weighted MR imaging of Apt-MNC solution was obtained using a 1.5-T clinical MR imaging instrument with a micro-47 surface coil (Intera, Philips Medical Systems, Andover, MA, USA) with the following parameters: resolution of 234 × 234 mm, section thickness of 2.0 mm, TE = 60 ms, TR = 4,000 ms, and number of acquisitions = 1. In addition, the relaxation rate (*R*2, unit of s^−1^) for various Fe concentrations of Apt-MNC was measured at room temperature by the Carr-Purcell-Meiboom-Gill sequence: TR = 10 s, 32 echoes, 12 ms even echo space, number of acquisitions = 1, point resolution 156 × 156 μm, and section thickness 0.6 mm.

### Biocompatibility tests for Apt-MNC

The cytotoxicity of Apt-MNC for U87MG cells (human glioblastoma) was evaluated by measuring the inhibition of cell growth using a 3-(4,5-dimethylthiazol-2-yl)-2,5-diphenyltetrazolium bromide (MTT) assay. U87MG cells (1.0 × 10^7^ cells) were plated in 96-well plates, incubated in MEM containing 5% fetal bovine serum and 1% antibiotics at 37°C in a humidified atmosphere with 5% CO_2_, and treated with carboxylated MNC or Apt-MNC at various concentrations for 24 h. An MTT assay was performed and the relative percentage of cell viability was calculated as the ratio of formazan intensity in cells treated with carboxylated MNC or Apt-MNC to the formazan intensity in non-treated cells.

### *In vitro* targeting assay

Sulfo-*N*-hydroxysuccinimide-modified fluorescein was purchased from Pierce® (fluorescein labeling kit, product no. 46100; Pierce Biotechnology, Rockford, IL, USA). To synthesize Apt-conjugated fluorescein (Apt-fluorescein), 0.067 mg of sulfo-*N*-hydroxysuccinimide-modified fluorescein and 0.5 mg Apt were mixed in RNAse-free water and incubated for 2 h at 4°C. After incubation, the mixture was purified with an ultracentrifugal filter (Amicon Ultra) to remove the side-products. We incubated 1.0 mmol of Apt-fluorescein with VEGFR2-expressing porcine aortic endothelial cells with overexpressing kinase insert domain receptor (PAE/KDR) cells (1.0 × 10^7^ cells) for 24 h at 37°C. The fluorescence-stained cells were detached and washed three times with PBS (pH 7.4, 1 mM). The cellular binding of Apt was evaluated via flow cytometry (Caliber, CA, USA) and visualized by confocal microscopy (LSM 700, Carl Zeiss AG, Oberkochen, Germany).

To evaluate the targeting affinity of Atp-MNC for VEGFR2 markers, 5.0 × 10^5^ PAE/KDR cells were seeded and incubated in four-well plates for 2 days at 37°C. Subsequently, the incubated cells were treated with Apt-MNC dispersed in DMEM and incubated for an additional 2 h at 37°C. The PAE/KDR cells treated with Apt-MNC were collected and washed two times with PBS. For observations of the attached Apt-MNC to the target marker, light-scattering images for cells were recorded using a microscope (Olympus BX51; Olympus Corporation, Tokyo, Japan) with a high numerical aperture dark-field condenser (U-DCW, Olympus), which delivers a very narrow beam of white light from a tungsten lamp to the surface of the sample. Immersion oil (nD 1.516, Olympus) was used to narrow the gap between the condenser and the glass slide and to balance the refractive index. The dark-field pictures were captured using an Olympus CCD camera [19].

### *In vivo* MR imaging

To establish the orthotopic brain tumor model, a sterilized guide screw was drilled in the skull of BALB/c nude mouse (4 to 6 weeks old) at an entry site with frontal lobe ordinates at 2 mm lateral and 1 mm anterior to the bregma. We implanted 5 × 10^5^ human glioblastoma U87MG cells suspended in 5 μL 2-[4-(2-hydroxyethyl)piperazin-1-yl] ethanesulfonic acid buffer onto the guide screw after 7 days of bolting. On the seventh day after implantation, the guide screw was removed and the incision was sutured. All experiments were conducted with the approval of the Association for Assessment and Accreditation of Laboratory Animal Care International [20].

MR imaging of the glioblastoma model treated with carboxylated MNC or Apt-MNC was performed with a 3.0-T MR imaging (Intera, Philips Medical Systems, *n* = 5). After intravenous injection into the tail vein using an insulin syringe (200 μg of Fe/200 μL), we performed *in vivo* imaging at various timed intervals. For *T*2-wieghted MR imaging, the following parameters were adopted: resolution of 234 × 234 mm, section thickness of 2.0 mm, TE = 60 ms, TR = 4,000 ms, and number of acquisitions = 1. Statistical evaluation of data was performed with analysis of variance test and Student's *t* test. A *p* value less than 0.01 was considered statistically significant.

### Histological analysis

Hematoxylin and eosin (H & E) staining and Prussian blue (PB) staining were performed for histological evaluation. The extracted brain tissue from mice injected with Apt-MNC was dehydrated in increasing alcohol concentrations, cleared in xylene, and embedded in paraffin. Tissue slices (thickness = 10 μm) were mounted on glass slides and were placed twice in a container filled with hematoxylin for 10 min to stain the nuclei. The tissues were rinsed in water for 10 min to remove hematoxylin, the cytoplasm was stained with eosin, and the samples were dehydrated in the same manner as described above. After washing three times for 30 min, we added 2 drops of the mounting solution onto the slide and covered it with a cover slip. To visualize the extent of Apt-MNC loading, an additional slide was fixed with 95% alcohol for 5 min, stained using a solution of 5% potassium ferrocyanide in 5% HCl (1:1) for 30 min at room temperature, and rinsed three times in deionized water to remove the residual staining solution. All tissue samples were analyzed using a research microscope (Olympus BX51) and OlyVIA software.

## Results and discussion

**Figure 2:**
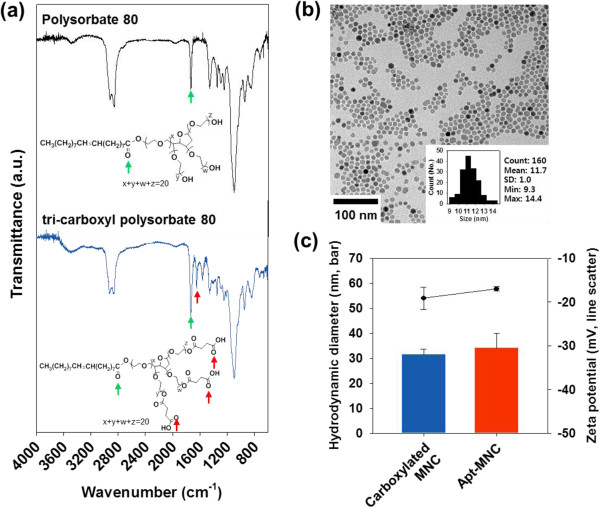
**Synthesis of Apt-MNC. (a)** FTIR spectrum of polysorbate 80 (black line) and tri-carboxyl polysorbate 80 (blue line). **(b)** TEM image of Apt-MNC (inset: size distribution histogram). **(c)** Hydrodynamic diameter (bar) and zeta potential (line scatter) of carboxylated MNC and Apt-MNC.

We synthesized high-quality MNC in terms of size uniformity, single crystallinity, and high magnetism, using the thermal decomposition method, for use as a sensitive MR imaging contrast agent [3]. The synthesized MNC exhibited water insolubility due to the presence of capped fatty acids; thus, this MNC should be modified using optimal surfactant to ensure its stability in biological media and biocompatibility *in vivo*. Here, carboxyl polysorbate 80 was prepared by modifying the hydroxyl group of polysorbate 80. Succinic anhydride reacted with the hydroxyl group on polysorbate 80 during the ring-opening process and the resultant terminal carboxylate was fabricated. The oxyethylene chains (-OCH_2_CH_2_-) in the carboxyl polysorbate 80 can increase biocompatibility, and carboxyl groups can be readily conjugated with the amine-functionalized targeting moieties [16]. After the ring-opening esterification reaction of polysorbate 80, the characteristic peaks of the modified carboxyl polysorbate 80 were confirmed by FTIR spectroscopy. In Figure 2a, polysorbate 80 and tri-carboxyl polysorbate 80 represented C=O stretching vibration at 1,737 cm^−1^ caused by ester structure (green arrow). However, the resultant terminal carboxylic acid in tri-carboxyl polysorbate 80 was confirmed by C=O stretching vibration at 1,652 cm^−1^ (red arrow). The dimer structure of carboxylic acid in a condensed undiluted solution weakened the C=O binding, thus C=O stretching vibration in carboxylic acid appeared to have a lower wave number than the C=O stretching vibration in ester.

Subsequently, MNC was coated by nanoemulsion method using carboxyl polysorbate 80. The nanoscale emulsions were created by the injection of MNC-laden organic solvent phase into an aqueous continuous phase containing carboxyl polysorbate 80 under ultrasonication and vigorous stirring. The interface of emulsions with continuum was stabilized by carboxyl polysorbate 80 and MNC within nanoemulsions and was enveloped by carboxyl polysorbate 80 during a solvent evaporation [17]. As described in the experimental section, Apt was conjugated with carboxylated MNC to prepare Apt-MNC for molecular MR imaging of VEGFR2. The morphology of Apt-MNC was observed by TEM. Uniformity and spherical shape of MNC from Apt-MNC were observed; the average diameter of MNC was 11.7 ± 1.0 nm and clustering of MNC was not observed (Figure 2b). The hydrodynamic diameter of Apt-MNC (34.0 ± 5.8 nm) was slightly increased compared with that of carboxylated MNC (31.5 ± 2.2 nm) due to Apt conjugation (Figure 2c). Carboxylated MNC possessed negative surface charge due to the negatively charged surface carboxylate in an aqueous phase. Apt-MNC showed a slightly changed surface charge of −17.0 ± 0.5 mV after Apt conjugation (Figure 2c). These data indicate that Apt was successfully conjugated with carboxylated MNC and Apt-MNC was well dispersed in an aqueous phase, with its monodispersity due to the presence of modified polysorbate 80 molecules. Additionally, negatively charged Apt-MNC surface repulsed nonspecific binding on negatively charged cell surface, increasing the aptamer-mediated specific binding on VEGFR2 [21]. Thus, the characteristics of Apt-MNC were suitable for a potential MR imaging probe to detect the biomarker.

**Figure 3:**
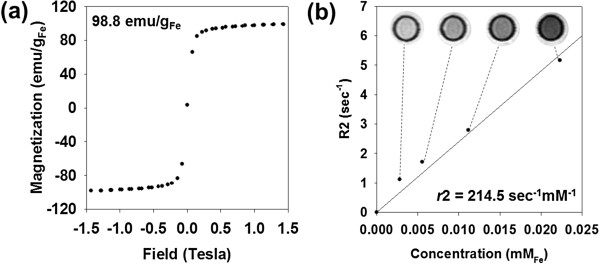
**Properties of Apt-MNC as a contrast agent. (a)** Magnetic hysteresis loop of Apt-MNC. **(b)***T*2-weighted images and relaxivity coefficient (*r*2) of Apt-MNC.

The prepared Apt-MNC exhibited a superparamagnetic property without magnetic hysteresis at zero magnetic field, and the saturation magnetization value was 98.8 emu g^−1^ Fe at 1.5 T. These magnetic properties were highly acceptable as a sensitive MR imaging contrast agent (Figure 3a). The *T*2-weighted MR imaging of Apt-MNC solution at various Fe concentrations was obtained to evaluate the capability of imaging contrast effect. The increase of Fe concentration accelerates transverse relaxation to shorten the *T*2 relaxation time (*T*2), resulting in a decreased signal intensity with dark contrast. The *T*2 relaxation rate (*R*2 = 1/*T*2, s^−1^) was plotted versus Fe concentration (mM) to determine the relaxivity coefficient (*r*2) as 214.5 s^−1^ mM^−1^, which is higher than that of commercial MR imaging contrast agents (ferumoxide, 190.5 s^−1^ mM^−1^) (Figure 3b) [22].

**Figure 4:**
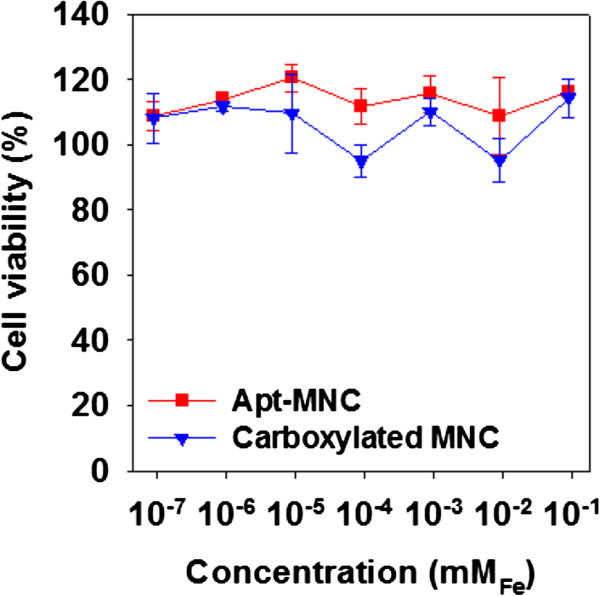
Cell viabilities of U87MG cells treated with different concentrations of Apt-MNC and carboxylated MNC.

To assess the biocompatibility of Apt-MNC, we investigated the *in vitro* cytotoxicity of carboxylated MNC and Apt-MNC in U87MG cells by monitoring the effects on cell viability and proliferation. Cell viabilities were examined after incubation with various concentrations of carboxylated MNC and Apt-MNC for 24 h. As shown in Figure 4, U87MG cells treated with each MNC solution showed no significant cytotoxicity effects even at high concentration (up to 5 × 10^−3^ mg Fe/mL). These results indicate that carboxylated MNC and Apt-MNC are biocompatible for use as MR imaging probes.

**Figure 5:**
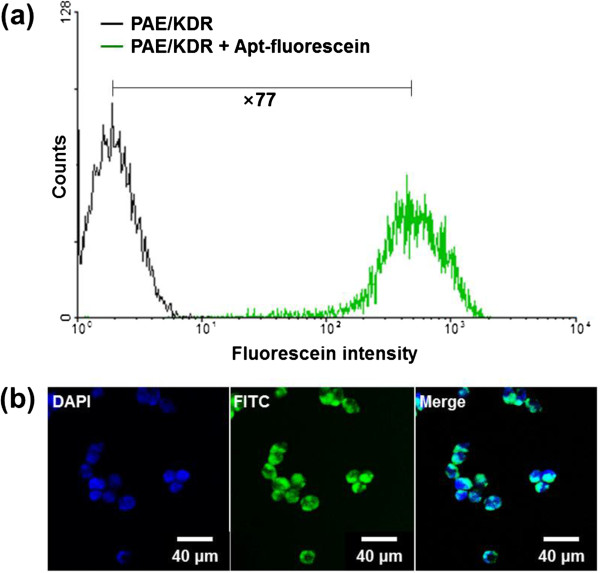
***In vitro *VEGFR2-targeting ability of Apt. (a)** Flow cytometry data of porcine aortic endothelial cells with overexpressing kinase insert domain receptor (PAE/KDR) cells treated with or without Apt-fluorescein. **(b)** Confocal microscopy images of PAE/KDR cells treated with DAPI (nucleus, blue) and Apt-fluorescein (cytoplasm, green).

**Figure 6:**
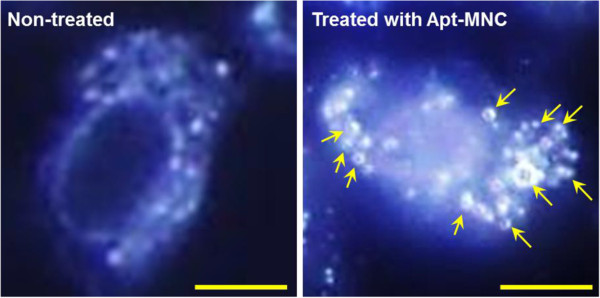
**Dark-field microscopy images of PAE/KDR cells.** (left panel) Non-treated and (right panel) treated with Apt-MNC; bars = 25 μm.

To assess the *in vitro* VEGFR2-targeting ability of Apt-MNC, VEGFR2-overexpressing PAE/KDR cells were treated with Apt-fluorescein, and the cells were analyzed by flow cytometry (Figure 5a). PAE/KDR cells treated with Apt-fluorescein exhibited fluorescence levels of 76.8% (green) when compared with that of non-treated PAE/KDR cells (control, 0.5% fluorescence level, black). PAE/KDR cells treated with Apt-fluorescein were analyzed by confocal microscopy (Figure 5b). Cells exhibited fluorescence in the nuclei (blue, DAPI) and in the cytoplasm (green, fluorescein); this confirmed that Apt could effectively bind to VEGFR2 expressed on PAE/KDR cells. The cellular binding efficiency of Apt-MNC was investigated using dark-field microscopy. In Figure 6, the scattered spots (yellow arrows) on PAE/KDR cells treated with Apt-MNC were observed due to MNC. However, carboxylate MNC without Apt conjugation was not observed in non-treated PAE/KDR cells. These results indicate that Apt-MNC effectively targeted VEGFR2-expressing cells [19].

**Figure 7:**
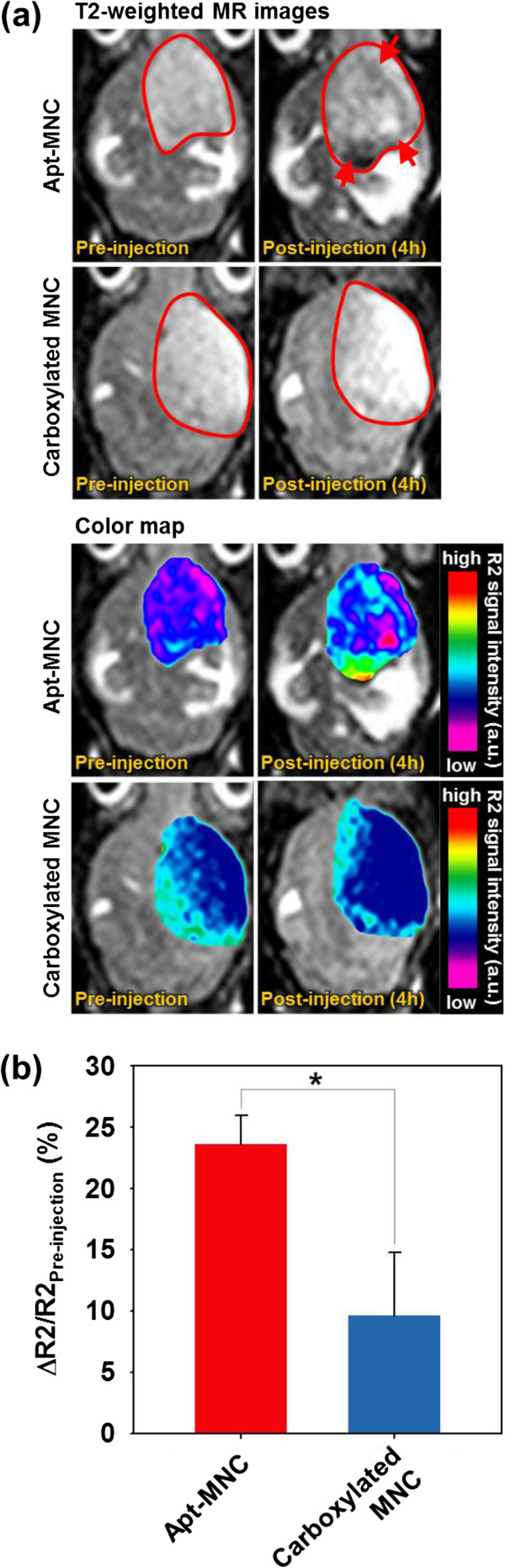
***In vivo *VEGFR2-targeting ability of Apt-MNC. (a)***T*2-weighted MR images and their color map for VEGFR2-expressing mouse model with intravenous injection of Apt-MNC or carboxylated MNC (red line: brain tumor, red arrow: contrast enhanced site). **(b)** Signal intensity graphs from *T*2-wieghted MR images (*p < 0.01).

To investigate *in vivo* VEGFR2-targeting ability of Apt-MNC using MR imaging, we prepared the glioblastoma-bearing mouse xenograft model by intracranial injection of U87MG cells into the brain. Although U87MG cancer cells did not express VEGFR2, they induced extensive VEGFR2 production through a tumor angiogenesis pathway when transplanted into mouse brain [20]. MR imaging for VEGFR2-expressing brain tumor was performed before and after intravenous injection of Apt-MNC and carboxylated MNC into the mouse tail vein (200 μg Fe), and the color map images of Apt-MNC and carboxylated MNC were presented to evaluate accurately the contrast change (Figure 7a). Before the administration of both MNC solutions (pre-injection), each *T*2-weighted MR image of the tumor site appeared characteristically bright with a low *R*2 value. Following injection of Apt-MNC or carboxylated MNC (postinjection), we observed that the tumor sites showed darkened images due to the presence of magnetic components. However, the tumor sites treated with Apt-MNC were darker than those treated with carboxylated MNC (red arrow) because Apt-MNC effectively targeted and bound to VEGFR2 of the tumor tissue, whereas carboxylated MNC was washed out in time [23-26]. In color map images, carboxylated MNC-treated mouse showed no color change in whole brain region (blue or cyan), but Apt-MNC-treated mouse showed significant color change in tumor site: violet (pre-injection) to green or red (postinjection). The MR imaging signal intensity (△*R*2/*R*2_pre-injection_; △*R*2 = *R*2 − *R*2_pre-injection_) of Apt-MNC-treated tumor sites was strongly enhanced, reaching a △*R*2/*R*2_pre-injection_ value of 23.6% after the injection (Figure 7b). However, as expected, when carboxylated MNC was administered to the mice, the △*R*2/*R*2_pre-injection_ values were 9.6% after injection, which were lower than half of the Apt-MNC signal intensity (*p* < 0.01). These MR imaging comparisons between Apt-MNC and carboxylated MNC confirmed that Apt effectively targets VEGFR2. Apt-MNC enabled the precise *in vivo* detection of VEGFR2 expressed in the glioblastoma model using MR imaging.

**Figure 8:**
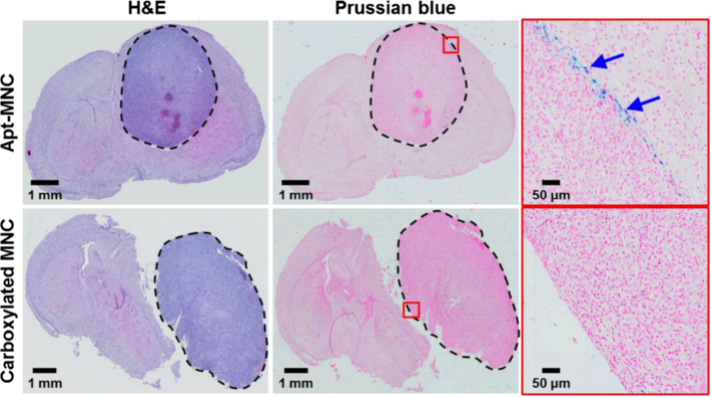
**Representative photographs of the brain stained with H & E and Prussian blue.** Representative photographs of the brain stained with H & E and Prussian blue after treated with Apt-MNC and carboxylated MNC. Ferric ions from Apt-MNC showed bright blue pigment (blue arrow).

To determine the precise regions detected by Apt-MNC, histological analysis was performed on the excised brain after nanoprobe treatment and MR imaging (Figure 8). The dark purple region in the H & E-stained tissues clearly outlined the tumor (first column). The selective accumulation of Apt-MNC within the tumor was verified using the Prussian blue staining kit (second column; third column, magnified images). Ferric ions from bound Apt-MNC in tumor tissue combined with the ferrocyanide and resulted in the formation of a bright blue pigment called Prussian blue (blue arrow). Tumor tissues treated with carboxylated MNC showed red (nuclei) and pink (cytoplasm) pigments, but lacked blue pigment. These results demonstrated that the tumor regions, which were identified in the *in vivo* MR imaging, were successfully targeted by Apt-MNC.

## Conclusions

We described the development of smart VEGFR2-targeting magnetic nanocrystal and evaluated its functional capability as a biomarker-detecting nanoprobe *in vitro* and *in vivo*. MNC was an ultrasensitive MR imaging contrast agent. MNC was synthesized using the thermal decomposition method, enveloped using biocompatible carboxyl polysorbate 80, and surface-modified using a VEGFR2-targetable aptamer. Apt-MNC exhibited a high magnetic resonance signal and efficient VEGFR2-detecting ability with no cytotoxicity. Consequently, selective targeting and high sensitivity in MR imaging contributed to the advantageous features of Apt-MNC as a novel VEGFR2-targeting nanoprobe. Furthermore, the incorporation of therapeutic agents in Apt-MNC might provide outstanding designs and applications for future clinical nanoprobes.

## Competing interests

The authors declare that they have no competing interests.

## Authors’ contributions

BK carried out the nanoparticle synthesis and modification and drafted the manuscript. JY conceived of the nanoparticle design and condition. MH carried out in vivo MR imaging. JC conceived of the design of the animal experiment. H-OK and EJ participated in the cellular targeting experiment. JHL and S-HR fabricated aptamer sequence. J-SS participated in the modification of magnetic resonance imaging sequence. Y-MH and SH participated in the design of whole study and drafted the manuscript. All authors read and approved the final manuscript.
